# Molecular detection of feline viruses in free-ranging European wildcats (*Felis s*. *silvestris*) of the Harz Mountains, Germany

**DOI:** 10.1007/s00705-026-06744-9

**Published:** 2026-09-21

**Authors:** Jutta Pikalo, Johanna Reiff, Martin Siegel, Peter Steinbach, Diana Jeschke, Ole Anders, Hermann Ansorge, Thomas W. Vahlenkamp, Mike Heddergott, Kristin Heenemann

**Affiliations:** 1 Institute of Parasitology, Department of Biological Sciences and Pathobiology, University of Veterinary Medicine Vienna, Veterinärplatz 1, Vienna, 1210 Austria; 2 Institute of Virology, Centre for Infectious Diseases, Faculty of Veterinary Medicine, Leipzig University, An den Tierkliniken 29, Leipzig, 04103 Germany; 3 Chair of Health Economics and Econometrics, University of Greifswald, Friedrich- Loeffler-Straße 70, Greifswald, 17489 Germany; 4 Faculty of Chemistry, University of Göttingen, Tammannstraße 4, Göttingen, 37077 Germany; 5 Musée National d’Histoire Naturelle, 25, rue Münster, Luxembourg, 2160 Luxembourg; 6 Senckenberg Museum of Natural History Görlitz, Am Museum 1, Görlitz, 02826 Germany; 7 Nationalpark Harz, Lindenallee 35, Werningerode, 38855 Germany; 8 International Institute Zittau, Technische Universität Dresden, Markt 23, Zittau, 02763 Germany

## Abstract

Many infectious diseases commonly affecting domestic cats may pose potential threats to wild felid populations. Several established contagious feline viruses — including feline leukemia virus (FeLV), feline immunodeficiency virus (FIV), feline coronavirus (FCoV), feline calicivirus (FCV), feline herpesvirus (FHV), and feline parvovirus (FPV) — are well-known drivers of disease epidemiology. In addition, recently identified agents such as feline circovirus (FeCV), feline morbillivirus (FeMV) and other novel paramyxoviruses as well as polyomaviruses may represent emerging risks to wild populations. We conducted a comprehensive molecular epidemiological survey to investigate the occurrence of these viruses in free-ranging European wildcats (*Felis s*. *silvestris*) from the Harz Mountains, a low mountain range in central Germany. A total of 42 individuals were sampled between 2010 and 2018. Molecular screening was performed using a comprehensive set of conventional PCR, reverse transcription PCR (RT-PCR), and quantitative RT-PCR (qRT-PCR) assays to detect viral nucleic acids. FeLV DNA was detected in 5/42 individuals (11.90%), and FPV DNA was detected in 2/42 wildcats (4.76%). Overall, viral nucleic acid was identified in seven animals (16.67%). No evidence of the remaining target viruses was found using the respective (q)RT-PCR and conventional PCR assays. However, negative PCR results should be interpreted with caution due to the exclusive use of long-term ethanol-preserved spleen samples, which may have reduced analytical sensitivity. The findings provide molecular evidence of FPV and FeLV, in free-ranging European wildcats in the Harz Mountains. Continued surveillance remains essential to better understand pathogen dynamics, evaluate potential spillover from domestic cats, and inform conservation strategies aimed at safeguarding wild felid populations in Central Europe.

**Supplementary Information:**

The online version contains supplementary material available at https://doi.org/10.1007/s00705-026-06744-9.

## Introduction

The European wildcat (*Felis s*. *silvestris*) represents a key mesocarnivore species within European forest ecosystems and is of high conservation concern in several parts of its distribution range with one major population group occurring in Germany [1]. Following drastic historical population declines throughout its European range, as well as in Germany, caused primarily by habitat loss, persecution, and hybridization with the domestic cat (*Felis catus*) - smaller, isolated populations remained in regions with large, contiguous forest areas, such as the Harz Mountains. From these small, isolated populations, the species has recolonized its former ranges thanks to legal protection measures and habitat restoration programs at both national and European levels [2, 3]. As population densities rise and spatial overlap with domestic cats increases, the risk of pathogen spillover at the wildlife–domestic animal interface has emerged as a possible critical issue in conservation medicine and disease ecology [4].

Viral pathogens are of particular relevance in this context, as several infectious agents known to affect domestic cats are capable of infecting free-ranging wildcats, potentially compromising individual fitness and long-term population viability [5–12]. Among the most extensively studied retroviruses are feline leukemia virus (FeLV), a gammaretrovirus, and feline immunodeficiency virus (FIV), a lentivirus. FeLV can induce immunosuppression, anemia, and neoplasia in domestic cats [5–11], and has been detected in European wildcat populations across multiple regions of Europe [13–20]. FeLV is shed in large quantities primarily in saliva, but can also be detected in urine, feces, and milk [21]. The virus survives only briefly outside the host due to its rapid inactivation by heat and desiccation [8, 21]. FeLV spreads through close contact between virus-shedding and naive cats and can also be transmitted vertically [21, 22]. The likelihood of developing a progressive FeLV infection decreases with increasing age [21]. FeLV prevalence in domestic cats varies considerably across Europe, with a north-south gradient and higher prevalence rates reported in Southern European countries [23, 24]. In Germany, an overall FeLV prevalence of 9.5% in domestic cats has been reported, although prevalence varies according to infection outcome and region [23–27]. FIV establishes lifelong infections associated with progressive immune dysfunction [6, 28, 29] and has likewise been reported in wildcat populations, with molecular evidence indicating both species-specific viral strains and possible cross-species transmission events [15, 29–31].

In addition to retroviruses, several common feline RNA viruses circulate at the wildlife–domestic animal interface. Feline coronavirus (FCoV), including variants associated with feline infectious peritonitis (FIP), have been identified in free-ranging wildcats, although its clinical significance in this species remains poorly understood [13, 14, 16–18, 32, 33]. Feline calicivirus (FCV) and feline herpesvirus (FHV) are widespread causes of upper respiratory tract disease in domestic cats [34–36] and have likewise been detected in European wildcat populations, indicating pathogen exchange in sympatric areas [13, 16–18]. Feline panleukopenia (FPL), a severe and often fatal disease affecting both domestic and wild felids, is caused by infection with members of the species *Protoparvovirus carnivoran 1* (PPVC 1), including feline parvovirus (FPV). Notably, all currently recognized variants of canine parvovirus (CPV-2a, CPV-2b, and CPV-2c) are capable of infecting cats, resulting in either subclinical infection or clinical signs consistent with FPL. These highly stable parvoviruses can induce severe enteritis and profound leukopenia [37–43].

More recently described viruses have expanded the spectrum of pathogens of potential concern. A novel feline circovirus (FeCV-1) was identified as the predominant circovirus in fecal samples from domestic cats in a study conducted in Italy [44], suggesting that FeCV-1 is a common component of the feline virome [45].

The family of paramyxoviruses has received increasing attention, with several new species recently identified. Notably, two distinct viral clusters have been identified in domestic cats: feline morbillivirus (FeMV), including the two main variants FeMV-1 and FeMV-2, and feline paramyxovirus (FePaV) [46]. Feline morbillivirus has been described in association with tubulointerstitial nephritis in domestic cats [47–49], while canine distemper virus (CDV), a related morbillivirus, can cause severe and often fatal disease in wild felids [50, 51]. FePaV was first identified in the kodkod (*Leopardus guigna*) in Chile [52], indicating its presence in wild felid populations. The extent to which these emerging viruses circulate in European wildcat populations, and whether they contribute to morbidity or mortality, remains largely unknown.

European populations of free-ranging wildcats have been shown to be exposed to cat viruses [13, 15–17, 53]. Understanding viral exposure patterns, infection dynamics, and clinical impacts in free-ranging European wildcats is essential for evidence-based conservation management. Given the increasing connectivity between wildlife and anthropogenic landscapes, systematic surveillance and comparative molecular analyses are required to clarify transmission pathways, assess the role of domestic cats as reservoirs, and evaluate the potential consequences of viral infections for the long-term viability of wildcat populations.

Accordingly, the aim of this study was to conduct a molecular-epidemiological investigation of a comprehensive set of feline viruses in a population of free-roaming European wildcats in the Harz Mountains, in order to record their prevalence, obtain baseline data on the circulation of pathogens, and contribute to the assessment of potential transmission risks at the interface between wild and domestic animals.

## Materials and methods

### Ethical statement

In accordance with Directive 2010/63/EU of the European Parliament and of the Council of 22 September 2010, on the protection of animals used for scientific purposes, no ethics approval was required for this type of study.

### Sample collection

**Fig. 1:**
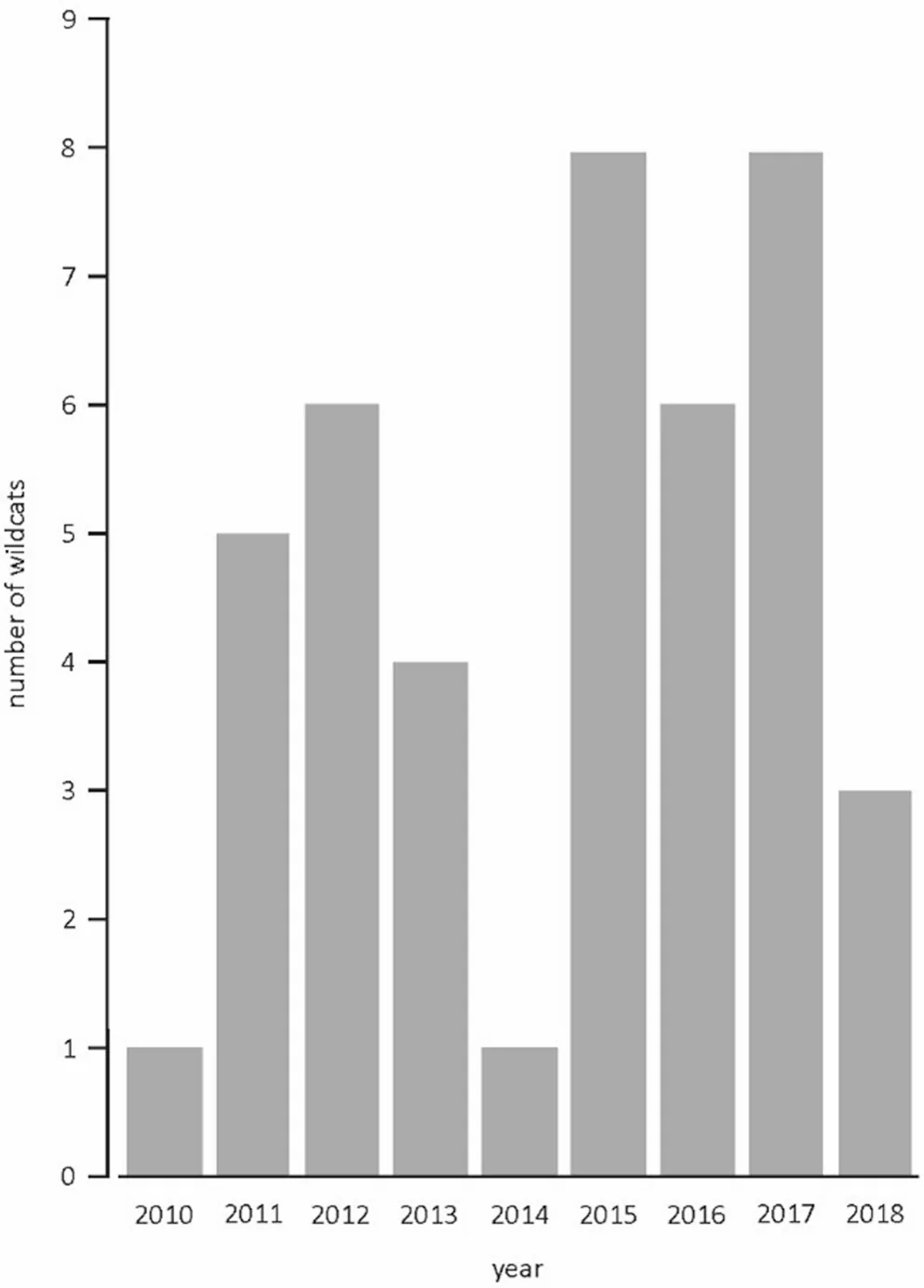
Distribution of the European wildcat (*Felis s. silvestris*) samples examined in this study, by year and number, from the Harz Mountains in Germany

 Between 2010 and 2018, 42 road-killed wildcats in the Harz Mountains in central Germany (Fig. 1) were recovered and stored at − 20 °C until necropsy. The collection was carried out as part of a monitoring program for animal carcasses by the Harz National Park and the state nature conservation authorities of the districts. Each individual was assigned a unique identification number and documented with respect to sex, age class, cause of death (e.g. traffic collision), and geographic location [17, 54]. The identification of the cats as wildcats was based on various morphological characteristics [55, 56], the cranial index [57], and the intestinal index [58]. During the necropsy at the Museum of Natural History of the Senckenberg Society for Nature Research in Görlitz, spleen samples were collected and stored in 70% ethanol at − 20 °C until transport and final processing at the University of Veterinary Medicine Vienna and the Faculty of Veterinary Medicine at the University of Leipzig. The samples examined in this study were the subject of earlier studies on vector-borne pathogens [54, 59]. Age determination was based on the condition of the dentition and measurements of the width of the dental pulp [60], with categorization following Piechocki and Stiefel [61] into the age classes juvenile (< 12 months), subadult (13–24 months), and adult (≥ 25 months). The resulting dataset comprised 26 males and 16 females, as well as 32 adults, 7 subadults, and 3 juveniles. The animals originated from three federal states: Lower Saxony (*n* = 31), Saxony-Anhalt (*n* = 4), and Thuringia (*n* = 7) (Table  S1).

### Viral RNA and DNA isolation, PCR amplification, and sequencing

Viral RNA and DNA were simultaneously extracted from spleen samples after overnight incubation at 56 °C with Proteinase K. The resulting supernatant was processed using the QIAamp 96 Virus QIAcube HT Kit (Qiagen, Hilden, Germany) on the QIAcube HT automated extraction system (Qiagen, Hilden, Germany), following the manufacturerˈs instructions. Following extraction, nucleic acid concentration and purity were assessed spectrophotometrically by measuring absorbance at 260 and 280 nm using a NanoDrop 2000c spectrophotometer (Thermo Fisher Scientific, Waltham, MA, USA). Negative controls were included in each extraction to monitor potential contamination.

The virological examination was conducted at the Institute of Virology, Faculty of Veterinary Medicine, Leipzig University. Eluates from the spleen were tested for the presence of key viral pathogens, including: FHV, FPV, FCoV, FCV, FeLV, and FIV.

Additionally, samples were also tested for circoviruses, polyomaviruses and paramyxoviruses. Detailed information on the (RT)-qPCR assays used are summarized in the following table (Table 1).

**Table 1:** Oligonucleotide sequences of primers and PCR-protocols used in the present study

Assay target	Primer sequences 5‘→3‘ and amplicon size	Temperature profile	Kit	Reference
Herpesviruses	**1. PCR**: KG1: 5’-GTCTTGCTCACCAGNTCNACNCCYTT-3’ DFA: 5’-GAYTTYGCNAGYYTNTAYCC-3’ ILK: 5’-TCCTGGACAAGCAGCARNYSGCNMTNAA-3’ **Amplicon size: ~700 bp** **2. PCR**: TGV: 5’-TGTACCTCGGTGTAYGGNTTYACNGGNGT-3’ IYG: 5’-CACAGAGTCCGTRTCNCCRTACAT-3’ **Amplicon size: ~250 bp**	**1.+2. PCR**: Initial denaturation: 95 °C 5 min Cycles (40x): 94 °C 30 s, 46 °C 30 s, 72 °C 30 s Final extension: 72 °C 7 min	Dream Taq DNA Polymerase (Thermo Scientific, Germany)	[62]
PPVC 1	M1: 5’-GAAAACGGATGGGTGGAAAT-3’ M2: 5’-AGTTGCCAATCTCCTGGATT-3’ **Amplicon size: 222 bp**	Initial denaturation: 95 °C 7 min Cycles (35x): 94 °C 30 s, 55 °C 30 s, 72 °C 30 s Final extension: 72 °C 10 min	Dream Taq DNA Polymerase (Thermo Scientific, Germany)	[63]
Coronaviruses	IZS-CoV forward: 5’-CDCAYGARTTYTGYTCNCARC-3’ IZS-CoV reverse: 5’-RHGGRTANGCRTCWATDGC-3’ **Amplicon size: 180 bp**	Initial denaturation: 60 °C 1 min RT-Step 45 °C 30 min Initial denaturation: 94 °C 2 min Cycles (45x): 94 °C 20 s, 45 °C 30 s, 68 °C 30 s Final extension: 68 °C 5 min	SuperScript^TM^III One-Step RT-PCR Platinum™ TaqHiFi (Invitrogen, Germany)	[64]
Caliciviruses	Calici-p289: 5’-TGACAATGTAATCATCACCATA-3’ Calici-p290: 5’-GATTACTCCAAGTGGGACTCCAC-3’ **Amplicon size: 331 bp**	Initial denaturation: 60 °C 1 min RT-Step 45 °C 30 min Initial denaturation: 94 °C 2 min Cycles (45x): 94 °C 20 s, 50 °C 30 s, 68 °C 30 s Final extension: 68 °C 5 min	SuperScript^TM^III One-Step RT-PCR Platinum™ TaqHiFi (Invitrogen, Germany)	[65]
FeLV	FeLV U3 s: 5’-TCAAGTATGTTCCCATGAGATACAA-3’ FeLV U3 as: 5’-GAAGGTCGAACTCTGGTCAACT-3’ **Amplicon size: 185 bp**	Initial denaturation: 95 °C 2 min Cycles (40x): 95 °C 5 s, 60 °C 10 s Melt 72–95 °C	QuantiNova SYBR Green PCR (Qiagen, Germany)	[66]
FIV	**1. PCR**: gag s: 5’-AAGAAATATTGGATGAAAGCCTAAAGC-3’ gag as: 5’-CAATTTTTCTAGGGTACTTTCTGG-3’ **Amplicon size: ~410 bp** **2. PCR**: gag nested s: 5’-CAAGATTTGCACCAGCTAGGATGC-3’ gag nested as: 5’-TGTTCTTGATCTATTTGGGC-3’ **Amplicon size: 168 bp**	**1.+2. PCR**: Initial denaturation: 94 °C 3 min Cycles (35x): 94 °C 30 s, 55 °C 30 s, 72 °C 30 s Final extension: 72 °C 10 min	Dream Taq DNA Polymerase (Thermo Scientific, Germany)	[67]
Circoviruses	**1. PCR**: Cv-s: 5’-AGAGGTGGGTCTTCACNHTBAAYAA-3’ Cv-as: 5’-AAGGCAGCCACCCRTARAARTCRTC-3’ **Amplicon size: ~600 bp** **2. PCR**: Cn-s: 5’-AGCAAGGAACCCCTCAYYTBCARGG-3’ Cn-as: 5’-ACGATGACTTCNGTCTTSMARTCACG-3’ **Amplicon size: 350 bp**	**1. PCR**: Initial denaturation: 95 °C 5 min Cycles (40x): 94 °C 30 s, 46 °C 30 s, 72 °C 30 s Final extension: 72 °C 7 min **2. PCR**: Initial denaturation: 95 °C 5 min Cycles (40x): 94 °C 30 s, 56 °C 30 s, 72 °C 30 s Final extension: 72 °C 5 min	Dream Taq DNA Polymerase (Thermo Scientific, Germany)	[68]
Polyomaviruses	**1. PCR**: Pv-s: 5’-CCAGACCCAACTARRAATGARAA-3’ Pv-as: 5’-AACAAGAGACACAAATNTTTCCNCC-3’ **Amplicon size: 829–1137 bp** **2. PCR**: Pn-s: 5’-ATGAAAATGGGGTTGGCCCNCTNTGYAARG-3’ Pn-as: 5’-CCCTCATAAACCCGAACYTCYTCHACYTG-3’ **Amplicon size: 249–273 bp**	**1. PCR**: Initial denaturation: 95 °C 5 min Cycles (40x): 94 °C 30 s, 46 °C 30 s, 72 °C 30 s Final extension: 72 °C 7 min **2. PCR**: Initial denaturation: 95 °C 5 min Cycles (40x): 94 °C 30 s, 56 °C 30 s, 72 °C 30 s Final extension: 72 °C 5 min	Dream Taq DNA Polymerase (Thermo Scientific, Germany)	[69]
Paramyxoviruses	**1. PCR**: PAR-F1: 5’-GAAGGXTATTGTCAXAARNTNTGGAC-3’ PAR-R: 5’-GCTGAAGTTACXGGXTCXCCDATRTTNC-3’ **Amplicon size: 662 bp** **2. PCR**: PAR-F2: 5’-GTTGCTTCAATGGTTCARGGNGAYAA-3’ PAR-R: 5’-GCTGAAGTTACXGGXTCXCCDATRTTNC-3’ **Amplicon size: 584 bp**	**1. PCR**: Initial denaturation: 60 °C 1 min RT-Step 45 °C 30 min Initial denaturation: 94 °C 2 min Cycles (45x): 94 °C 15 s, 48 °C 30 s, 68 °C 30 s Final extension: 68 °C 5 min **2. PCR**: Initial denaturation: 94 °C 2 min Cycles (45x): 94 °C 15 s, 48 °C 20 s, 72 °C 30 s Final extension: 72 °C 5 min	SuperScript^TM^III One-Step RT-PCR Platinum™ TaqHiFi (Invitrogen™, Germany)	[70]

Positive and negative controls were included in all (RT)-qPCR assays to validate assay performance and monitor for contaminations.

PCR products were analyzed by electrophoresis in 2% agarose gels stained with Midori Green Advance DNA stain (Nippon Genetics Europe, Germany). Amplicons from positive samples were purified using the Thermo Scientific GeneJET PCR Purification Kit (Thermo Fisher Scientific, Germany) according to the manufacturer’s instructions to remove unincorporated primers, nucleotides, and enzymes prior to sequencing. Purified products were subsequently sent to Microsynth Seqlab GmbH (Göttingen, Germany), for Sanger sequencing using amplification primers in both directions.

The resulting sequences were assembled using the BioEdit software, version 7.7.1 (North Carolina State University, Raleigh, USA) and compared with sequences available in NCBI GenBank (National Center for Biotechnology; GenBank (https://blast.ncbi.nlm.nih.gov/Blast.cgi*).*

### Statistical analysis

Data were recorded and evaluated using Microsoft Excel 365 (Microsoft Corporation, Redmond, WA, USA) and STATA 19.0 (Stata Corp. 2025. TX, USA) was used for all statistical analyses. The 95% Confidence interval (CI) was calculated according to Clopper and Pearson [71]. A χ^2^-test was performed to assess associations between the presence of viral nucleic acids and sex, age, and location. Logistic regression was used to evaluate the effects of sex, age, and location category on the presence of viral nucleic acids. Effects were considered statistically significant if *p* < 0.05.

## Results

### Detection of viral genetic material

At least one virus was detected in 7 of 42 wildcats (16.7%; 95% CI 5.4–27.9). Feline leukemia virus was detected in 5 of 42 (11.9%; 95% CI 4.0–25.4) samples. Feline parvovirus was detected in 2 of 42 (4.8%; 95% CI 0.6–16.2) wildcats. Only single viral infections were detected. No viral DNA or RNA from the following pathogens was detected: FHV, FCoV, FCV, FIV, circoviruses, polyomaviruses, or paramyxoviruses (Table  S1).

The obtained sequences were deposited in the NCBI GenBank Database (National Center for Biotechnology Information) with following accession numbers: PZ239367 - PZ239373.

The molecular characteristics of virus-positive individuals are summarized in Table 2. All obtained sequences showed high nucleotide identity (96–100%) to the respective reference sequences (Table 2).

**Table 2:** Molecular characteristics of virus-positive European wildcats (*Felis s*. *silvestris*) from Harz Mountains in Germany

Sample ID	Virus	GenBank accession number	Identity to reference strain* (%)	Location	Sex	Age	Year
20_2155	FPV	PZ239367	100.00	Lower Saxony	m	adult	2018
32_2268	FPV	PZ239368	100.00	Thuringia	m	adult	2012
40_2284	FeLV	PZ239373	96.15	Lower Saxony	f	adult	2016
36_2277	FeLV	PZ239372	96.15	Lower Saxony	f	adult	2016
29_2263	FeLV	PZ239371	96.70	Lower Saxony	m	adult	2016
22_2159	FeLV	PZ239370	96.15	Lower Saxony	m	adult	2016
17_2147	FeLV	PZ239369	96.70	Saxony-Anhalt	m	adult	2011

*f* female, *m* male; *Reference Strain FPV: AY742937.1; FeLV: NC_001940.1

**Fig. 2:**
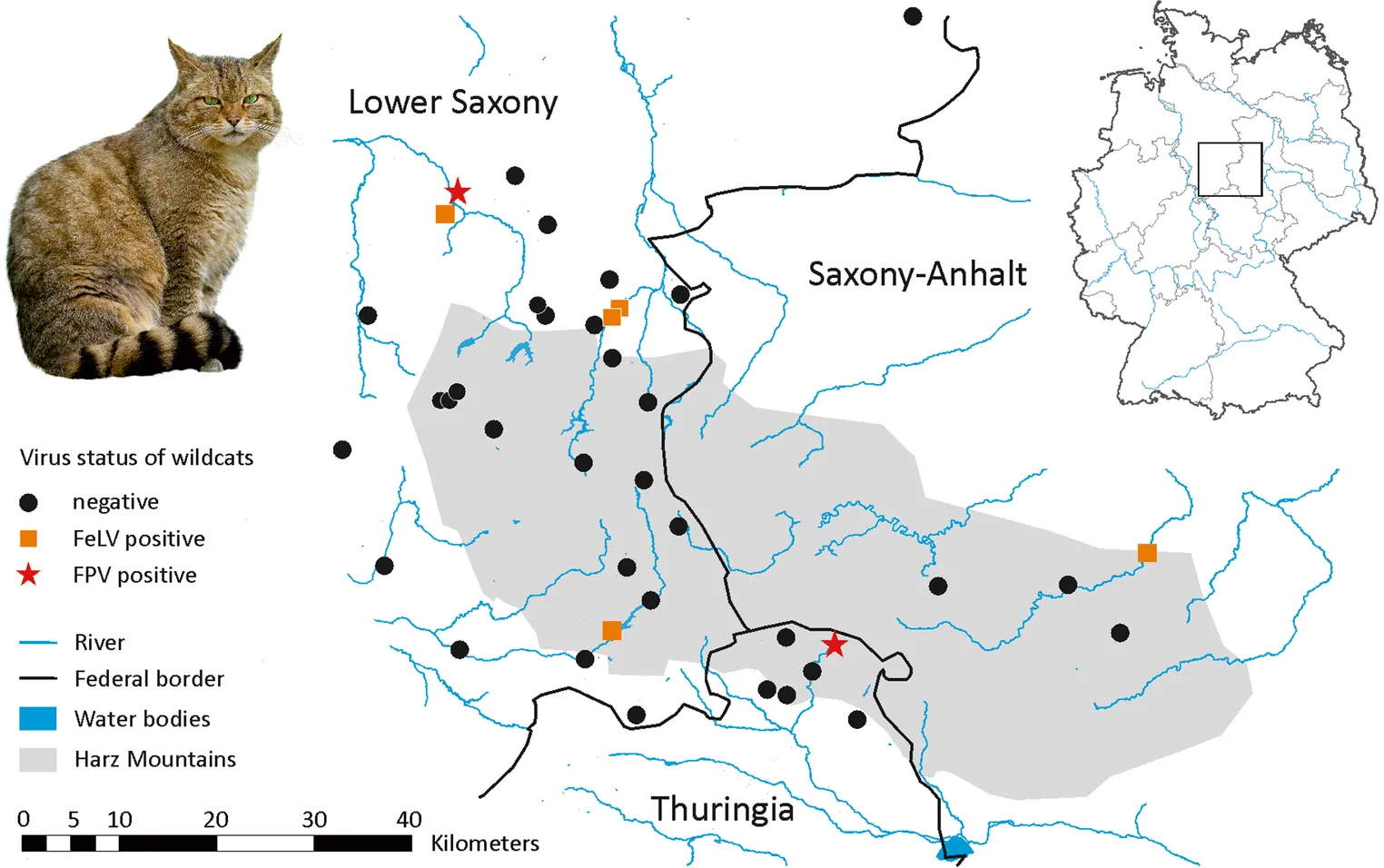
Geographical distribution of the 42 European wildcats (*Felis s*. *silvestris*) from Harz Mountains in Germany included in this study

The geographic distribution of the virus-positive individuals as well as the negative tested animals are shown in Fig. 2.

No statistically significant associations were found between the sexes for either FeLV or FPV (FeLV: *p* = 0.926; FPV: *p* = 0.256) or age groups (FeLV: *p* = 0.412; FPV: *p* = 0.720), nor were there any significant associations based on state of origin (FeLV: *p* = 0.443; FPV: *p* = 0.415). Owing to the limited number of wildcats examined, the analyses had low statistical power and may have been insufficient to detect existing associations.

## Discussion

In this first molecular epidemiological study of free-ranging European wildcats from the Harz Mountains, in Germany, viral nucleic acids were detected in seven of 42 individuals, with positive findings limited to FeLV and FPV. Because detection relied solely on nucleic acid screening, findings reflect molecular evidence of a pathogen but do not confirm active viral replication, the presence of infectious virus, or virus-associated disease. Molecular screening did not identify additional viral targets among the investigated pathogens. These findings provide further insight into pathogen circulation in a protected wild felid species and highlight the relevance of domestic cat-associated viruses at the wildlife-domestic animal interface.

It is noteworthy that FeLV DNA was detected in 11.9% of the wildcats examined, since a previous serological study of wildcats from the Harz Mountains using the SNAP^®^ FIV/FeLV Combo Test did not detect any antigen of FeLV [17]. This is significant because Heddergott et al. [17] reported a decline in the prevalence of FeLV antigens from south to north, and the present study provides molecular evidence of FeLV in the wildcat population of the Harz Mountains.

In European wildcats, FeLV infection has been reported sporadically, often in areas with sympatric domestic cat populations [12–20, 53, 72]. Our results corroborate previous molecular evidence of FeLV in free-ranging wildcats in Central Europe. Although interspecific transmission from domestic cats is biological plausible in regions with habitat overlap, the present data do not allow conclusions regarding the source or direction of transmission.

Spillover of FeLV from domestic cats to wild felids has been repeatedly documented, sometimes with severe conservation consequences, most prominently in the Iberian lynx (*Lynx pardinus*), the Florida panther (*Puma concolor coryi*) or in the North American bobcats (*Lynx rufus*) [73–77]. This should be taken into account, especially when reintroducing endangered species into new areas particularly if they have not been vaccinated [78]. Given the potential for persistent infection and population-level effects, even moderate prevalence rates warrant continued surveillance.

According to the Standing Committee on Vaccination in Veterinary Medicine (StIKo Vet) [27] and the European Advisory Board on Cat Diseases (ABCD) [79] vaccination is recommended for domestic cats at high risk of exposure, such as those with outdoor access or those in contact with cats of unknown FeLV status [27, 79]. Therefore, the prevalence of FeLV infection has decreased in domestic cats in Europe over the last years [8]. Compared with previous studies, the potential transmission to wild felids may also be reduced.

In this study, only adult animals tested positive for FeLV proviral DNA, resulting in an observed prevalence of 11.90%. Detection of FeLV proviral DNA indicates previous or ongoing infection but does not allow conclusions regarding active viral replication or infection outcome. The observed prevalence is comparable to that reported in wildcats from other countries [13, 19, 20, 53], although higher FeLV prevalences have been reported in Luxembourg, Portugal, France, and Southwest Germany [12, 14–16, 18, 80]. The interpretation of these comparisons should take into account the considerable variation in sample size and the test systems among studies. The present findings further support the higher sensitivity of molecular detection compared with rapid antigen testing, as previously reported [17, 81]. Therefore, we recommend using molecular biological assays for RNA/DNA detection instead of rapid tests for antigen detection in future studies. Furthermore, marked differences in sampling duration and the geographic extent of the surveyed areas may affect the comparability of the findings.

Feline parvovirus nucleic acid was detected in two individuals (4.76%). This is the first genetic evidence of FPV in wildcats from the Harz Mountains. Heddergott et al. [17] previously reported the presence of antibodies against FPV in wildcats from this region. Members of this viral species, including FPV and canine protoparvovirus variants, are characterized by high environmental stability after shedding of high virus titers in the feces and therefore efficient transmission also via contaminated objects or people [38, 43, 82]. In domestic cats, systemic infection of various tissues and organ systems, may result in feline panleukopenia, a disease associated with high morbidity and mortality, particularly in juveniles if the cats are not vaccinated [43]. Canids, raccoons (*Procyon lotor*), American mink (*Neogale vison*) and red foxes (*Vulpes vulpes*) can also be infected and thus act as further hosts for the virus [82]. However, in the present study, all positive individuals were adults, suggesting that subclinical or transient infections may occur in older animals [14, 18]. Molecular and serological evidence of parvovirus exposure has also been described in wild felids, including European wildcats [12, 14, 16–18, 50, 53, 82–85]. The comparatively low detection rate in the present study could reflect subclinical infections, transient viral shedding, sampling bias toward clinically healthy individuals, reduced analytical sensitivity associated with sample age and storage conditions, or limited statistical power due to sample size or immunization of domestic cats. Immunization against feline panleukopenia is classified as a core vaccination in domestic cats in Germany. Continuous protective immunity is recommended against disease caused by FPV, with vaccines also providing cross-protection against circulating variants of PPVC 1. Owing to the consistent implementation of routine vaccination programs, the disease is currently well controlled in domestic cat populations in Germany, which may reduce opportunities for transmission to wild carnivores [27]. Thus, the presence of FPV supports the assumption that environmentally resistant parvoviruses are maintained in multi-host systems involving domestic and wild carnivores.

Although sex was not significantly associated with virus positivity, five of the seven virus-positive wildcats were male. In addition to environmental transmission, territorial and aggressive interactions particularly among adult males during the mating season, could potentially facilitate the transmission of viruses spread through close contact [53, 86]. However, the small number of positive animals precludes any firm conclusions.

In contrast, no nucleic acids of FIV, FCoV, FCV, FHV, FeCV, FeMV, FePaV, or polyomaviruses were detected, although antibodies against FCV, FCoV, and FHV have been found in wildcats in this area [17]. Some of these pathogens or antibodies against the viruses have been identified in domestic cats and, in some cases, in wild felids [12–18, 30, 33, 49, 52, 53, 85]. Their apparent absence in our dataset may indicate low prevalence in the studied population or limited sensitivity due to sample type and timing of infection. Particularly for viruses characterized by intermittent shedding or tissue tropism not represented in the analyzed material, (RT)q PCR-based screening of single time-point samples may underestimate true exposure. The suitability of spleen tissue for molecular detection differs among the investigated viruses according to their tissue tropism and shedding patterns. For FHV and FCV, respiratory, conjunctival, and/or oropharyngeal samples are commonly used for molecular detection, whereas FCoV is primarily an enteric virus and is shed in feces, although systemic infection can involve various tissues [32, 35, 36, 87]. Feline circoviruses have also been detected in fecal samples [44, 45], whereas feline morbilliviruses and other feline paramyxoviruses have been associated with renal tissue [46–49]. Furthermore, Lyon-IARC polyomavirus (LIPyV) has been detected in fecal samples from domestic cats [88, 89]. Thus, the exclusive examination of spleen tissue may have reduced the likelihood of detecting some of the investigated viruses.

Moreover, nucleic acids may degrade during long-term storage and therefore escape detection. Antibodies against FCV, FCoV, and FHV remain detectable for longer periods and have been identified, indicating previous exposure to these pathogens [17]. These findings highlight the importance of combining molecular and serological approaches to obtain a more comprehensive assessment of pathogen exposure and infection status.

Spleen samples were stored in 70% ethanol at − 20 °C for extended periods, with a median age of 12 years (range: 8–18 years) at the time of extraction. This storage method is not optimal for long-term nucleic acid preservation, as prolonged storage in ethanol at − 20 °C may reduce the recovery of amplifiable nucleic acids over time [90–92]. Given the advanced age of the samples, the observed low detection rates may reflect not only low true prevalence but also reduced sensitivity due to nucleic acid degradation. Therefore, the absence of viral nucleic acids in some tested pathogens should be interpreted with caution, as it may be influenced by sample quality rather than true absence. Not all molecular assays used in this study have been specifically validated for tissues from European wildcats, which represents an additional limitation when interpreting the absence of detectable viral nucleic acids.

Given the small sample size and the limited number of virus-positive animals, the statistical analyses should be considered exploratory, and the absence of significant associations should be interpreted with caution.

The findings of this study are relevant in light of the reintroduction of the Eurasian lynx (*Lynx lynx*) in the Harz Mountains, which was launched in 2000 by the Lower Saxony Ministries of Agriculture and Nature Conservation, as well as the Lower Saxony State Hunting Association, which served as the project’s sponsoring organizations [93]. Genetic monitoring of wildcat species is routinely conducted using hair traps equipped with valerian-treated bait sticks [94, 95]. Felids frequently leave scent marks at prominent landscape features by urinating [96] or depositing feces [97]; previous studies have also shown that the attractant sticks at these stations are often sprayed with urine by the animals that visit them [98]. In addition to urine marking, felids communicate chemically via secretions from facial and tail glands—which are transferred by rubbing against objects—as well as through scratching behavior, which leaves both visual and olfactory signals on tree bark and other substrates [97, 99, 100].

Because FPV exhibits considerable environmental persistence, these marking behaviors may facilitate contamination of lure sticks and thereby create opportunities for indirect virus transmission among cats. Furthermore, FPV can be mechanically transported on contaminated fomites, including footwear and clothing [43], raising the possibility that field personnel could inadvertently disseminate the virus between sampling locations. In regions where FPV is endemic, biosecurity measures should therefore be implemented to reduce transmission risk. Such measures may include the use of disposable lure sticks, thorough disinfection of reusable equipment, and periodic relocation of lure stations within the study area [17]. Furthermore, a high prevalence of feline viral diseases could have a negative impact on lynx populations. It is also known that wildcats [101] and domestic cats [102] are occasionally preyed upon by lynx.

From a conservation perspective, our findings underscore the importance of pathogen monitoring in fragmented and recovering European wildcat populations. Habitat overlap, hybridization events, and anthropogenic landscape changes may increase contact rates between wildcats and domestic cats, potentially creating opportunities for pathogen transmission [103, 104]. Long-term surveillance integrating molecular, serological, and ecological data will be essential to elucidate transmission dynamics, evaluate potential consequences for host health and population dynamics and support targeted management strategies. In particular, monitoring FeLV and FPV at the wildlife-domestic interface should be prioritized to mitigate potential health threats to wildcat populations in Central Europe.

## Supplementary Information

Below is the link to the electronic supplementary material.

Supplementary Material 1

## Data Availability

All data supporting the findings of this study are available within the paper and its Supplementary Information.

## References

[1] Mattucci F, Oliveira R, Lyons LA, Alves PC, Randi E (2015) European wildcat populations are subdivided into five main biogeographic groups: consequences of Pleistocene climate changes or recent anthropogenic fragmentation? Ecol Evol 6(1):3–22. 10.1002/ece3.1815 PMC4716505 26811770

[2] Kitchener AC, Breitenmoser-Würsten C, Breitenmoser U (2025) European Wildcat *Felis silvestris* Schreber, 1777, and African Wildcat *Felis lybica* Forster, 1780. In: Hackländer K, Zachos FE (eds) Handbook of the Mammals of Europe. Handbook of the Mammals of Europe. Springer, Cham. 10.1007/978-3-319-65038-8_122-1

[3] European Topic Centre on Biological Diversity (2019) Article 17 web tool on biogeographical assessments of conservation status of species and habitats under Article 17 of the Habitats Directive. Europäische Umweltagentur https://nature-art17.eionet.europa.eu/article17/

[4] Poirson C, Dutilleul S (2014) Coordination Mammalogique du Nord de la France, pour le Conseil Régional Nord-Pas de Calais; Plan régional de restauration du chat forestier (*Felis silvestris*) et de la martre des pins (*Martes martes*) en Nord-Pas de Calais

[5] Hofmann-Lehmann R, Fehr D, Grob M, Elgizoli M, Packer C, Martenson JS, O’Brien SJ, Lutz H (1996) Prevalence of antibodies to feline parvovirus, calicivirus, herpes-virus, coronavirus, and immunodeficiency virus and of feline leukemia virus antigen and the interrelationship of these viral infections in free-ranging lions in east Africa. Clin Diagn Lab Immunol 3:554–562. 10.1128/cdli.3.5.554-562.1996 PMC170405 8877134

[6] Hosie MJ, Addie D, Belák S, Boucraut-Baralon C, Egberink H, Frymus T, Gruffydd-Jones T, Hartmann K, Lloret A, Lutz H, Marsilio F, Pennisi MG, Radford AD, Thiry E, Truyen U, Horzinek MC (2009) Feline immunodeficiency. ABCD guidelines on prevention and management. J Feline Med Surg 11(7):575–584. 10.1016/j.jfms.2009.05.006 PMC7129779 19481037

[7] Lutz H, Isenbügel E, Lehmann R, Sabapara RH, Wolfensberger C (1992) Retrovirus Infections in non-domestic felids: Serological studies and attempts to isolate a lentivirus. Vet Immunol Immunopathol 35:215–224. 10.1016/0165-2427(92)90133-b 1337398

[8] Lutz H, Addie D, Belák S, Boucraut-Baralon C, Egberink H, Frymus T, Gruffydd-Jones T, Hartmann K, Hosie MJ, Lloret A, Marsilio F, Pennisi MG, Radford AD, Thiry E, Truyen U, Horzinek MC (2009) Feline leukaemia. ABCD guidelines on prevention and management. J Feline Med Surg 11(7):565–574. 10.1016/j.jfms.2009.05.005 PMC7172531 19481036

[9] Olmsted RA, Langley R, Roelke ME, Goeken RM, Adger-Johnson D, Goff JP, Albert JP, Packer C, Laurenson MK, Caro TM, Scheepers L, Wildt DE, Bush M, Martenson JS, O’Brien SJ (1992) Worldwide prevalence of lentivirus infection in wild feline species: Epidemiologic and phylogenetic aspects. J Virol 66:6008–6018. 10.1128/jvi.66.10.6008-6018.1992 PMC241478 1382145

[10] Paul-Murphy J, Work T, Hunter D, McFie E, Fjelline D (1994) Serologic survey and serum biochemical reference ranges of the free-ranging mountain lion (*Felis concolor*) in California. J Wildl Dis 30:205–215. 10.7589/0090-3558-30.2.205 8028105

[11] Spencer JA (1991) Survey of antibodies to feline viruses in free-ranging lions. S Afr J Wildl Res 21:59–61

[12] Stubbe SP, Lang J, Nagler N, Müller SF, Lierz M (2025) High prevalence of antigens of and specific antibodies against various viral pathogens in European wildcats (*Felis silvestris*) from Southwest Germany, 2020-22. J Wildl Dis 61(3):663–673. 10.7589/JWD-D-24-00191 40420390

[13] Daniels MJ, Golder MC, Jarett O, MacDonald DW (1999) Feline viruses in wildcats from Scotland. J Wildl Dis 35:121–124. 10.7589/0090-3558-35.1.121 10073361

[14] Duarte A, Fernandes M, Santos N, Tavares L (2012) Virological survey in free-ranging wildcats (*Felis silvestris*) and feral domestic cats in Portugal. Vet Microbiol 158(3–4):400–404. 10.1016/j.vetmic.2012.02.033 PMC7117533 22424865

[15] Fromont E, Sager A, Leger F, Bourgemestre F, Jouquelet E, Stahl P, Pontier D, Artois M (2000) Prevalence and pathogenicity of retroviruses in wildcats in France. Vet Rec 146:317–319. 10.1136/vr.146.11.317 10766116

[16] Heddergott M, Steeb S, Osten-Sacken N, Steinbach P, Schneider S, Pir JP, Müller F, Pigneur LM, Frantz AC (2018) Serological survey of feline viral pathogens in free-living European wildcats (*Felis s. silvestris*) from Luxembourg. Arch Virol 163:3131–3134. 10.1007/s00705-018-3972-x PMC7087253 30062638

[17] Heddergott M, Pikalo J, Müller F, Steinbach P, Wittische J, Steeb S, Jeschke D, Anders O, Ansorge H, Frantz AC (2026) Exposure to Feline viruses in European wildcats (*Felis s. silvestris*) in Germany: Spatial patterns and environmental risk factors. Viruses 18:627. 10.3390/v18060627 PMC13307804 42357636

[18] Leutenegger CM, Hofmann-Lehmann R, Riols C, Liberek M, Worel G, Lups P, Fehr D, Hartmann M, Weilenmann P, Lutz H (1999) Viral infections in free-living populations of the European wildcat. J Wildl Dis 35:678–686. 10.7589/0090-3558-35.4.678 10574526

[19] McOrist S, Boid R, Jones TW, Easterbee N, Hubbard AL, Jarrett O (1991) Some viral and protozool diseases in the European wildcat (*Felis silvestris*). J Wildl Dis 27:693–696. 10.7589/0090-3558-27.4.693 1758037

[20] McOrist S (1992) Diseases of the European wildcat (*Felis silvestris* Schreber, 1777) in Great Britain. Rev Sci Tech 11:1143–1149. 10.20506/rst.11.4.655 1339067

[21] Hofmann-Lehmann R, Hartmann K (2020) Feline leukaemia virus infection: A practical approach to diagnosis. J Feline Med Surg 22(9):831–846. 10.1177/1098612X20941785 PMC11135663 32845225

[22] Hartmann K, Hofmann-Lehmann R (2020) What’s new in Feline leukemia virus infection. Vet Clin North Am Small Anim Pract 50(5):1013–1036. 10.1016/j.cvsm.2020.05.006 32680664

[23] Giselbrecht J, Jähne S, Bergmann M, Meli ML, Pineroli B, Boenzli E, Teichmann-Knorrn S, Zablotski Y, Pennisi MG, Layachi N, Serra R, Bo S, Hofmann-Lehmann R, Hartmann K (2023) Prevalence of different courses of Feline leukaemia virus Infection in four European countries. Viruses 15(8):1718. 10.3390/v15081718 PMC10459464 37632060

[24] Studer N, Lutz H, Saegerman C, Gönczi E, Meli ML, Boo G, Hartmann K, Hosie MJ, Moestl K, Tasker S, Belák S, Lloret A, Boucraut-Baralon C, Egberink HF, Pennisi MG, Truyen U, Frymus T, Thiry E, Marsilio F, Addie D, Hochleithner M, Tkalec F, Vizi Z, Brunetti A, Georgiev B, Ludwig-Begall LF, Tschuor F, Mooney CT, Eliasson C, Orro J, Johansen H, Juuti K, Krampl I, Kovalenko K, Šengaut J, Sobral C, Borska P, Kovaříková S, Hofmann-Lehmann R (2019) Pan-European study on the prevalence of the Feline leukaemia virus infection - Reported by the European Advisory Board on Cat Diseases (ABCD Europe). Viruses 11(11):993. 10.3390/v11110993 PMC6893802 31671816

[25] Englert T, Lutz H, Sauter-Louis C, Hartmann K (2012) Survey of the feline leukemia virus infection status of cats in Southern Germany. J Feline Med Surg 14(6):392–398. 10.1177/1098612X12440531 PMC10822583 22403413

[26] Gleich SE, Krieger S, Hartmann K (2009) Prevalence of feline immunodeficiency virus and feline leukaemia virus among client-owned cats and risk factors for infection in Germany. J Feline Med Surg 11(12):985–992. 10.1016/j.jfms.2009.05.019 PMC11318771 19616984

[27] StIKo Vet (Standing Committee on Vaccination in Veterinary Medicine) (2025) Guideline on vaccination of small animals 6th edition. OpenAgrar. https://www.openagrar.de/servlets/MCRFileNodeServlet/openagrar_derivate_00063989/Impfleitlinie_Kleintiere_2025-01-06.pdf Accessed 10 April 2026

[28] Beatty J (2014) Viral causes of feline lymphoma: retroviruses and beyond. Vet J 201(2):174–180. 10.1016/j.tvjl.2014.05.026 24928422

[29] White J, Stickney A, Norris JM (2011) Feline immunodeficiency virus: disease association versus causation in domestic and nondomestic felids. Vet Clin North Am Small Anim Pract 41(6):1197–1208. 10.1016/j.cvsm.2011.07.003 22041211

[30] Courchamp F, Yoccoz NG, Artois M, Pontier D (1998) At-risk individuals in Feline Immunodeficiency Virus epidemiology: evidence from a multivariate approach in a natural population of domestic cats (*Felis catus*). Epidemiol Infect 121(1):227–236. 10.1017/s0950268898008875 PMC2809496 9747777

[31] Troyer JL, Pecon-Slattery J, Roelke ME, Johnson W, VandeWoude S, Vazquez-Salat N, Brown M, Frank L, Woodroffe R, Winterbach C, Winterbach H, Hemson G, Bush M, Alexander KA, Revilla E, O’Brien SJ (2005) Seroprevalence and genomic divergence of circulating strains of feline immunodeficiency virus among Felidae and Hyaenidae species. J Virol 79(13):8282–8294. 10.1128/JVI.79.13.8282-8294.2005 PMC1143723 15956574

[32] Addie D, Belák S, Boucraut-Baralon C, Egberink H, Frymus T, Gruffydd-Jones T, Hartmann K, Hosie MJ, Lloret A, Lutz H, Marsilio F, Pennisi MG, Radford AD, Thiry E, Truyen U, Horzinek MC (2009) Feline infectious peritonitis ABCD guidelines on prevention and management. J Feline Med Surg 11(7):594–604. 10.1016/j.jfms.2009.05.008 PMC7129471 19481039

[33] Watt NJ, MacIntyre NJ, McOrist S (1993) An extended outbreak of infectious peritonitis in a closed colony of European wildcats (*Felis silvestris*). J Comp Pathol 108(1):73–79. 10.1016/s0021-9975(08)80229-0 PMC7130280 8386199

[34] Gaskell RM, Dawson S (1994) Viral-induced upper respiratory tract disease. In: Chandler EA, Gaskell CJ, Gaskell RM (eds) Feline Medicine and Therapeutics. Blackwell Scientific, Oxford, pp 453–472

[35] Radford AD, Addie D, Belák S, Boucraut-Baralon C, Egberink H, Frymus T, Gruffydd-Jones T, Hartmann K, Hosie MJ, Lloret A, Lutz H, Marsilio F, Pennisi MG, Thiry E, Truyen U, Horzinek MC (2009) Feline calicivirus infection. ABCD guidelines on prevention and management. J Feline Med Surg 11(7):556–564. 10.1016/j.jfms.2009.05.004 PMC11132273 19481035

[36] Thiry E, Addie D, Belák S, Boucraut-Baralon C, Egberink H, Frymus T, Gruffydd-Jones T, Hartmann K, Hosie MJ, Lloret A, Lutz H, Marsilio F, Pennisi MG, Radford AD, Truyen U, Horzinek MC (2009) Feline herpesvirus infection. ABCD guidelines on prevention and management. J Feline Med Surg 11(7):547–555. 10.1016/j.jfms.2009.05.003 PMC7129359 19481034

[37] Battilani M, Bassani M, Forti D, Morganti L (2006) Analysis of the evolution of feline parvovirus (FPV). Vet Res Commun 30(Suppl 1):223–226. 10.1007/s11259-006-0046-4

[38] Miranda C, Thompson G (2016) Canine parvovirus: the worldwide occurrence of antigenic variants. J Gen Virol 97(9):2043–2057. 10.1099/jgv.0.000540 27389721

[39] Barrs VR (2019) Feline panleukopenia: A re-emergent disease. Vet Clin North Am Small Anim Pract 49(4):651–670. 10.1016/j.cvsm.2019.02.006 30967253

[40] Stuetzer B, Hartmann K (2014) Feline parvovirus infection and associated diseases. Vet J 201(2):150–155. 10.1016/j.tvjl.2014.05.02 24923754

[41] Truyen U, Evermann JF, Vieler E, Parrish CR (1996a) Evolution of canine parvovirus involved loss and gain of feline host range. Virology 215:186–189. 10.1006/viro.1996.0021 8560765

[42] Truyen U, Evermann JF, Vieler E, Parrish CR (1996b) Evolution of the feline–subgroup parvovirus and the control of canine host range in vivo. J Virol 63:4702–4710. 10.1128/jvi.69.8.4702-4710.1995 PMC189276 7609035

[43] Truyen U, Addie D, Belák S, Boucraut-Baralon C, Egberink H, Frymus T, Gruffydd-Jones T, Hartmann K, Hosie MJ, Lloret A, Lutz H, Marsilio F, Pennisi MG, Radford AD, Thiry E, Horzinek MC (2009) Feline panleukopenia. ABCD guidelines on prevention and management. J Feline Med Surg 11(7):538–546. 10.1016/j.jfms.2009.05.002 PMC7129762 19481033

[44] Vasinioti VI, Pellegrini F, Buonavoglia A, Capozza P, Cardone R, Diakoudi G, Desario C, Catella C, Vicenza T, Lucente MS, Di Martino B, Camero M, Elia G, Decaro N, Martella V, Lanave G (2023) Investigating the genetic diversity of CRESS DNA viruses in cats identifies a novel feline circovirus and unveils exposure of cats to canine circovirus. Res Vet Sci 161:86–95. 10.1016/j.rvsc.2023.06.011

[45] Vasinioti VI, Salvaggiulo A, Pellegrini F, Diakoudi G, Camero M, Mesquita JR, Gomes-de-Sá S, Buzdugan I, Savuţa G, Elia G, Decaro N, Martella V, Lanave G (2025) Feline circovirus-1 in domestic cats: A multicentric European epidemiological study. Res Vet Sci 196:105920. 10.1016/j.rvsc.2025.105920 41046711

[46] Sieg M, Busch J, Eschke M, Böttcher D, Heenemann K, Vahlenkamp A, Reinert A, Seeger J, Heilmann R, Scheffler K, Vahlenkamp TW (2019) A new genotype of feline morbillivirus infects primary cells of the lung, kidney, brain and peripheral blood. Viruses 11(2):146. 10.3390/v11020146 PMC6410220 30744110

[47] Pennisi MG, Belák S, Tasker S, Addie DD, Boucraut-Baralon C, Egberink H, Frymus T, Hartmann K, Hofmann-Lehmann R, Lloret A, Marsilio F, Thiry E, Truyen U, Möstl K, Hosie MJ (2023) Feline morbillivirus: Clinical relevance of a widespread endemic viral infection of cats. Viruses 15:2087. 10.3390/v15102087 PMC10611265 37896864

[48] Sieg M, Heenemann K, Rückner A, Burgener I, Oechtering G, Vahlenkamp TW (2015) Discovery of new feline paramyxoviruses in domestic cats with chronic kidney disease. Virus Genes 51:294–297. 10.1007/s11262-015-1232-7 26265247

[49] Woo PC, Lau SK, Wong BH, Fan RY, Wong AY, Zhang AJ, Wu Y, Choi GK, Li KS, Hui J, Wang M, Zheng BJ, Chan KH, Yuen KY (2012) Feline morbillivirus, a previously undescribed paramyxovirus associated with tubulointerstitial nephritis in domestic cats. Proc Natl Acad Sci U S A 109(14):5435–5440. 10.1073/pnas.1119972109 PMC3325679 22431644

[50] Candela MG, Pardavila X, Ortega N, Lamosa A, Mangas JG, Martínez-Carrasco C (2019) Canine distemper virus may affect European wild cat populations in Central Spain. Mamm Biol 97(1):9–12. 10.1016/j.mambio.2019.04.006 PMC7091739 32218716

[51] Roelke-Parker ME, Munson L, Packer C, Kock R, Cleaveland S, Carpenter M, O’Brien SJ, Pospischil A, Hofmann-Lehmann R, Lutz H, Mwamengele GL, Mgasa MN, Machange GA, Summers BA, Appel MJ (1996) A canine distemper virus epidemic in Serengeti lions (*Panthera leo*). Nature 379:441–445. 10.1038/379441a0 PMC7095363 8559247

[52] Sieg M, Sacristán I, Busch J, Terio KA, Cabello J, Hidalgo-Hermoso E, Millán J, Böttcher D, Heenemann K, Vahlenkamp TW, Napolitano C (2020) Identification of novel feline paramyxoviruses in Guignas (*Leopardus guigna*) from Chile. Viruses 12(12):1397. 10.3390/v12121397 PMC7762136 33291219

[53] Millán J, Rodriguez A (2009) A serological survey of common feline pathogens in free-living European wildcats (*Felis silvestris*) in Central Spain. Eur J Wildl Res 55:285–291. 10.1007/s10344-008-0246-z PMC7087923 32214938

[54] Unterköfler MS, Harl J, Barogh BS, Spergser J, Hrazdilová K, Müller F, Jeschke D, Anders O, Steinbach P, Ansorge H, Fuehrer HP, Heddergott M (2022) Molecular analysis of blood-associated pathogens in European wildcats (*Felis silvestris silvestris*) from Germany. Int J Parasitol Parasites Wildl 19:128–137. 10.1016/j.ijppaw.2022.08.012 PMC9477852 36119442

[55] Müller F (2005) Zur Diagnostik von Wild- und Hauskatze (Felis silvestris und F. catus, Felidae) nach morphologischen und anatomischen Merkmalen. Beitr Naturkunde Osthess 41:9–18

[56] Müller F (2011) Körpermerkmale als Unterscheidungskriterien zwischen wildfarbenen Hauskatzen (F. s. catus) und Wildkatzen (Felis s. silvestris, Felidae) aus Mitteleuropa. Beitr Jagd Wildforsch 36:359–368

[57] Schauenberg P (1969) L’identification du chat forestier d’Europe *Felis s. silvestris* Schreber, 1777, par une m´ethode ost´eom´etrique. Revue suisse de Zool 76:441–453 5394218

[58] Braunschweig A (1963) Untersuchungen an Wildkatzen und diesen ähnlichen Hauskatzen. Z Jagdwiss 9:109–112

[59] Panait LC, Mihalca AD, Modrý D, Juránková J, Ionică AM, Deak G, Gherman CM, Heddergott M, Hodžić A, Veronesi F, Reichard M, Zieman EA, Nielsen CK, Jiménez-Ruiz FA, Hrazdilová K (2021) Three new species of *Cytauxzoon* in European wild felids. Vet Parasitol 290:109344. 10.1016/j.vetpar.2021.109344 33465567

[60] Götz M (2015) Altersbestimmung anhand odontologischer Merkmale von Wildkatzen. Methoden und Ergebnisse des Totfundmonitorings in Sachsen-Anhalt. Beitr Jagd Wildforsch 40:19–30

[61] Piechocki R, Stiefel A (1988) Über die Altersstruktur der Verluste der Wildkatze (*Felis s. silvestris* Schreber 1777). Hercynia NF pp. 235–258

[62] Van Devanter DR, Warrener P, Bennett L, Schultz ER, Coulter S, Garber RL, Rose TM (1996) Detection and analysis of diverse herpesviral species by consensus primer PCR. J Clin Microbiol 34(7):1666–1671. 10.1128/JCM.34.7.1666-1671.1996 PMC229091 8784566

[63] Frölich K, Streich WJ, Fickel J, Jung S, Truyen U, Hentschke J, Dedek J, Prager D, Latz N (2005) Epizootiologic investigations of parvovirus infections in free-ranging carnivores from Germany. J Wildl Dis 41(1):231–235. 10.7589/0090-3558-41.1.231 15827228

[64] Lelli D, Papetti A, Sabelli C, Rosti E, Moreno A, Boniotti MB (2013) Detection of coronaviruses in bats of various species in Italy. Viruses 5(11):2679–2689. 10.3390/v5112679 PMC3856409 24184965

[65] Martella V, Pinto P, Lorusso E, Di Martino B, Wang Q, Larocca V, Cavalli A, Camero M, Decaro N, Bányai K, Saif LJ, Buonavoglia C (2015) Detection and full-length genome characterization of novel canine vesiviruses. Emerg Infect Dis 21(8):1433–1436. 10.3201/eid2108.140900 PMC4517720 26196075

[66] Langhammer S, Hübner J, Kurth R, Denner J (2006) Antibodies neutralizing feline leukaemia virus (FeLV) in cats immunized with the transmembrane envelope protein p15E. Immunology 117(2):229–237. 10.1111/j.1365-2567.2005.02291.x PMC1782217 16423059

[67] Vahlenkamp TW (1993) Untersuchungen zur Wirksamkeit antiviraler Mittel gegenüber der felinen Immunschwächevirus (FIV)-Infektion in in vitro-Testsystemen und an experimentell FIV-infizierten Katzen. Dissertation. Ludwig-Maximilians-University, Munich, Germany

[68] Halami MY, Nieper H, Müller H, Johne R (2008) Detection of a novel circovirus in mute swans (*Cygnus olor*) by using nested broad-spectrum PCR. Virus Res 132(1–2):208–212. 10.1016/j.virusres.2007.11.001 18082907

[69] Johne R, Enderlein D, Nieper H, Müller H (2005) Novel polyomavirus detected in the feces of a chimpanzee by nested broad-spectrum PCR. J Virol 79(6):3883–3887. 10.1128/JVI.79.6.3883-3887.2005 PMC1075742 15731285

[70] Tong S, Chern SW, Li Y, Pallansch MA, Anderson LJ (2008) Sensitive and broadly reactive reverse transcription-PCR assays to detect novel paramyxoviruses. J Clin Microbiol 46(8):2652–2658. 10.1128/JCM.00192-08 PMC2519498 18579717

[71] Clopper CJ, Pearson ES (1934) The use of confidence or fiducial limits illustrated in the case of the binomial. Biometrika 26(4):404–413. 10.1093/biomet/26.4.404

[72] Didkowska A, Matusik K, Klich D, Schmidt K, Kwiecień E, Wódz K, Kwieciński P, Wójcik W, Anusz K, Kołodziej-Sobocińska M (2025) The prevalence of highly-pathogenic viruses in European wildcat and Eurasian Lynx in Poland. Vet Res Commun 50(1):34. 10.1007/s11259-025-10971-x PMC12619723 41240245

[73] Geret CP, Cattori V, Meli ML, Riond B, Martínez F, López G, Vargas A, Simón MA, López-Bao JV, Hofmann-Lehmann R, Lutz H (2011) Feline leukemia virus outbreak in the critically endangered Iberian lynx (*Lynx pardinus*): high-throughput sequencing of envelope variable region A and experimental transmission. Arch Virol 156(5):839–854. 10.1007/s00705-011-0925-z 21302124

[74] López G, López-Parra M, Fernández L, Martínez-Granados C, Martínez F, Meli ML, Gil-Sánchez JM, Viqueira N, Díaz-Portero MA, Cadenas R, Lutz H, Vargas, A Simón MA (2009) Management measures to control a feline leukemia virus outbreak in the endangered Iberian lynx. Anim Conserv 12:173–182. 10.1111/j.1469-1795.2009.00241.x

[75] Meli ML, Cattori V, Martínez F, López G, Vargas A, Simón MA, Zorrilla I, Muñoz A, Palomares F, López-Bao JV, Pastor J, Tandon R, Willi B, Hofmann-Lehmann R, Lutz H (2009) Feline leukemia virus and other pathogens as important threats to the survival of the critically endangered Iberian lynx (*Lynx pardinus*). PLoS ONE 4(3):e4744. 10.1371/journal.pone.0004744 PMC2649436 19270739

[76] Meli ML, Cattori V, Martínez F, López G, Vargas A, Palomares F, López-Bao JV, Hofmann-Lehmann R, Lutz H (2010) Feline leukemia virus infection: a threat for the survival of the critically endangered Iberian lynx (*Lynx pardinus*). Vet Immunol Immunopathol 134(1–2):61–67. 10.1016/j.vetimm.2009.10.010 PMC7127500 19896221

[77] Petch RJ, Gagne RB, Chiu E, Mankowski C, Rudd J, Roelke-Parker M, Vickers TW, Logan KA, Alldredge M, Clifford D, Cunningham MW, Onorato D, VandeWoude S (2022) Feline leukemia virus frequently spills over from domestic cats to North American pumas. J Virol 96(23):e0120122. 10.1128/jvi.01201-22 PMC9749473 36374109

[78] Nájera F, Grande-Gómez R, Peña J, Vázquez A, Palacios MJ, Rueda C, Corona-Bravo AI, Zorrilla I, Revuelta L, Gil-Molino M, Jiménez J (2021) Disease surveillance during the reintroduction of the Iberian Lynx (*Lynx pardinus*) in Southwestern Spain. Anim (Basel) 11(2):547. 10.3390/ani11020547 PMC7923217 33669869

[79] ABCD 2026 European Advisory Board on Cat Diseases https://www.abcdcatsvets.org/. Accessed 10 April 2026

[80] Steeb S (2015) Postmortale Untersuchungen an der Europäischen Wildkatze (*Felis silvestris silvestris* Schreber, 1777). DVM Dissertation, Department of Veterinary Medicine, Justus-Liebig-Universität Gießen, Germany

[81] Nájera F, López G, Del Rey-Wamba T, Malik RA, Garrote G, López-Parra M, Fernández-Pena L, García-Tardío M, Arenas-Rojas R, Simón MA, Zorrilla I, Fernández I, Alcaide EM, Ruiz C, Revuelta L, Salcedo J, Hofmann-Lehmann R, Meli ML (2024) Long-term surveillance of the feline leukemia virus in the endangered Iberian lynx (*Lynx pardinus*) in Andalusia, Spain (2008–2021). Sci Rep 14(1):5462. 10.1038/s41598-024-55847-3 PMC10914683 38443503

[82] Steinel A, Parrish CR, Bloom ME, Truyen U (2001) Parvovirus infections in wild carnivores. J Wildl Dis 37(3):594–607. 10.7589/0090-3558-37.3.594 11504234

[83] Blanco K, Peña R, Hernández C, Jiménez M, Araya LN, Romero JJ, Dolz G (2011) Serological detection of viral infections in captive wild cats from Costa Rica. Vet Med Int 1:879029. 10.4061/2011/879029 PMC3087557 21547230

[84] Canuti M, Mira F, Villanúa D, Rodríguez-Pastor R, Guercio A, Urra F, Millán J (2025) Molecular ecology of novel amdoparvoviruses and old protoparvoviruses in Spanish wild carnivorans. Infect Genet Evol 128:105714. 10.1016/j.meegid.2025.105714 39809349

[85] Ostrowski S, Van Vuuren M, Lenain DM, Durand A (2003) A serologic survey of wild felids from central west Saudi Arabia. J Wildl Dis 39(3):696–701. 10.7589/0090-3558-39.3.696 14567233

[86] Hellard E, Fouchet D, Santin-Janin H, Tarin B, Badol V, Coupier C, Leblanc G, Poulet H, Pontier D (2011) When cats’ ways of life interact with their viruses: a study in 15 natural populations of owned and unowned cats (*Felis silvestris catus*). Prev Vet Med 101(3–4):250–264. 10.1016/j.prevetmed.2011.04.020 21705099

[87] Hofmann-Lehmann R, Hosie MJ, Hartmann K, Egberink H, Truyen U, Tasker S, Belák S, Boucraut-Baralon C, Frymus T, Lloret A, Marsilio F, Pennisi MG, Addie DD, Lutz H, Thiry E, Radford AD, Möstl K (2022) Calicivirus infection in cats. Viruses 14(5):937. 10.3390/v14050937 PMC9145992 35632680

[88] Fahsbender E, Altan E, Estrada M, Seguin MA, Young P, Leutenegger CM, Delwart E (2019) Lyon-IARC Polyomavirus DNA in feces of diarrheic cats. Microbiol Resour Announc 8(29):e00550–e00519. 10.1128/MRA.00550-19 PMC6639616 31320414

[89] Li Y, Huang H, Lan T, Wang W, Zhang J, Zheng M, Cao L, Sun W, Lu H (2021) First detection and complete genome analysis of the Lyon IARC polyomavirus in China from samples of diarrheic cats. Virus Genes 57(3):284–288. 10.1007/s11262-021-01840-1 PMC8107205 33970402

[90] Barnes I, Holton J, Vaira D, Spigelman M, Thomas MG (2000) An assessment of the long-term preservation of the DNA of a bacterial pathogen in ethanol-preserved archival material. J Pathol 192(4):554–559. 10.1002/1096-9896(2000)9999:9999<::AID-PATH768>3.0.CO;2-C 11113876

[91] Oosting T, Hilario E, Wellenreuther M, Ritchie PA (2020) DNA degradation in fish: Practical solutions and guidelines to improve DNA preservation for genomic research. Ecol Evol 10:8643–8651. 10.1002/ece3.6558 PMC7452763 32884647

[92] Zimmermann J, Hajibabaei M, Blackburn DC, Hanken J, Cantin E, Posfai J, Evans TC Jr (2008) DNA damage in preserved specimens and tissue samples: a molecular assessment. Front Zool 5:18. 10.1186/1742-9994-5-18 PMC2579423 18947416

[93] Anders O, Middelhoff TL (2021) The development of the Harz lynx population. CATnews Special Issue 14:24–28

[94] Schmidt K, Kowalczyk R (2006) Using scent-marking stations to collect hair samples to monitor Eurasian lynx populations. Wildl Soc Bull 34:462–466. 10.2193/0091-7648(2006)34[462:USSTCH]2.0.CO;2

[95] Steyer K, Simon O, Kraus RH, Haase P, Nowak C (2013) Hair trapping with valerian-treated lure sticks as a tool for genetic wildcat monitoring in low-density habitats. Eur J Wildl Res 59:39–46. 10.1007/s10344-012-0644-0

[96] Ruiz-Olmo J, Such-Sanz A, Piñol C (2013) Substrate selection for urine spraying in captive wildcats. J Zool 290:143–150. 10.1111/jzo.12025

[97] Piñeiro A, Barja I (2015) Evaluating the function of wildcat faecal marks in relation to the defence of favourable hunting areas. Ethol Ecol Evol 27:161–172. 10.1080/03949370.2014.905499

[98] Velli E, Bologna MA, Silvia C, Ragni B, Randi E (2015) Non-invasive monitoring of the European wildcat (*Felis silvestris silvestris* Schreber, 1777): Comparative analysis of three different monitoring techniques and evaluation of their integration. Eur J Wildl Res 61:657–668. 10.1007/s10344-015-0936-2

[99] Feldman HN (1994) Methods of scent marking in the domestic cat. Can J Zool 72:1093–1099

[100] Mohorović M, Krofel M (2020) The scent world of cats: Where to place a urine scent mark to increase signal persistence? Anim Biol 71:151–168. 10.1163/15707563-bja10018

[101] Müller F (2016) Luchs (*Lynx lynx*) tötet Wildkatze (*Felis silvestris*). Säugeierkdl Inf 51:237–243

[102] Nájera F, Sánchez-Cuerda S, López G, Del Rey-Wamba T, Rueda C, Vallverdú-Coll N, Panadero J, Palacios MJ, López-Bao JV, Jiménez J (2019) Lynx eats cat: disease risk assessment during an Iberian lynx intraguild predation. Eur J Wildl Res 65(3):39. 10.1007/s10344-019-1275-5 PMC7087927 32214947

[103] Lecis R, Pierpaoli M, Birò ZS, Szemethy L, Ragni B, Vercillo F, Randi E (2006) Bayesian analyses of admixture in wild and domestic cats (*Felis silvestris*) using linked microsatellite loci. Mol Ecol 15(1):119–131. 10.1111/j.1365-294X.2005.02812.x 16367835

[104] Randi E, Pierpaoli M, Beaumont M, Ragni B, Sforzi A (2001) Genetic identification of wild and domestic cats (*Felis silvestris*) and their hybrids using Bayesian clustering methods. Mol Biol Evol 18(9):1679–1693. 10.1093/oxfordjournals.molbev.a003956 11504848

